# Scarlet fever: a guide for general practitioners

**DOI:** 10.1080/17571472.2017.1365677

**Published:** 2017-08-11

**Authors:** S. Basetti, J. Hodgson, T. M. Rawson, A. Majeed

**Affiliations:** a School of Medicine, Imperial College London, London, UK; b Department of Primary Care and Public Health, Imperial College London, London, UK; c Health Protection Research Unit in Healthcare Associated Infections and Antimicrobial resistance, Imperial College London, London, UK

**Keywords:** Scarlet fever, pharyngitis, macropapular rash, tonsillar swab

## Abstract

There has been an increase in the incidence of scarlet fever with most cases presenting in General Practice and Emergency Departments. Cases present with a distinctive macro-papular rash, usually in children. This article aims to increase awareness of scarlet fever by highlighting key symptoms and stating potential complications if untreated. In patients who have the typical symptoms, a prescription of a suitable antibiotic such as phenoxymethylpenicillin (Penicillin V) should be made immediately to reduce the risk of complications and the spread of infection.

## Why this matters to me

There has been an increase in the incidence of Scarlet Fever presenting to general practice and emergency departments across the U.K. This has led Public Health England to issue an alert to health practitioners to be mindful of this diagnosis when assessing patients. We hope to support this message. This article aims to emphasise the importance of rapid diagnosis and treatment to prevent onward transmissions and potential complications such as spesis, abcesses and acute rheumatic fever.

## Key message

Early diagnosis by recognising the ‘tell-tale’ signs of scarlet fever could help reduce the risk of complications and prevent further spread, especially in children.

## Background

Scarlet fever or ‘scarlatina’ is the name given to a disease caused by an infective Group A Streptococcal (GAS) bacteria. It usually presents as exudative pharyngitis with a spreading maculo-papular rash originating from the trunk [1]. For many years, the incidence of scarlet fever was declining. However, there has been a recent increase in the number of cases worldwide. During the last decade, there have been major reported outbreaks in a number of countries. For example, in Asia, Vietnam reported over 23,000 cases and mainland China reported over 100,000 cases [2] in 2009. Smaller outbreaks have also occurred in USA and Canada. In the U.K., Public Health England has reported a total of 12,906 cases between September 2015 and April 2016, the largest outbreak in the U.K. since 1969 [3].

## Typical presentation

A typical presentation of scarlet fever is highlighted below:

An 8-year-old girl is brought to you by their parents at to your practice. She has a sore throat, abdominal pain and has been vomiting. Her general health has been fine until two days ago. Initially, she noticed pain on swallowing and had a temperature of 38 °C. Her parents decided to take her to the GP once they noticed the ‘sandpaper-like’ rash on her trunk and the inside of her elbows. When you ask to examine her tongue, the enlarged papillae become immediately obvious giving it a ‘strawberry’ like appearance. Petechiae are also visible on her soft palate. Her anterior-cervical nodes are swollen and tender.

As in this case, scarlet fever typically presents with high fevers, an erythematous sore throat, strawberry-like tongue and a sand-paper like rash. This rash almost always originates from the groin and spreads bilaterally up the trunk to the axilla, at 7–10 days the rash spreads to the extremities and desquamates. Desquamation can be noted only on the palms and soles, not the trunk [4]. There are no signs of upper respiratory inflammation, differentiating scarlet fever from measles and rubella [5].

Although scarlet fever can affect any age, due to its preponderance in children aged between 3 and 8 years, it is important to be extra vigilant when reviewing children in this age group. The incidence of scarlet fever among males is typically higher than females. Surges in scarlet fever incidence have been repeatedly reported during the winter and summer months [6].

Many of the presenting symptoms associated with scarlet fever are similar to those caused by other common infections in this age group, such as infection with Epstein-Barr virus, adenovirus or other respiratory viruses. Scarlet fever is mainly a clinical diagnosis. Diagnostic aids such as the Centor Score (modified McIsaac Score) [1] can be used to help guide diagnosis (Table 1). The Centor Score comprises four clinical signs and symptoms, which are used to estimate the probability of GAS pharyngitis. When using this score, the likelihood of a streptococcus infection is reasonably specific when ≥3 signs or symptoms are present (0.82) and very specific when ≥4 are present (0.95) [7]. The performance of the score is robust and it performs consistently across different healthcare settings in a variety of countries [7].

**Table 1.** A table showing how the Centor Score is calculated due to the presence of certain clinical signs and how they correlate towards estimating post-test risk of infection [7].

Symptoms	Points	Score	Post-test probability (%)
Tonsillar exudates	1	0	2.5
Tender anterior cervical adenopathy	1	1	6.5
Absence of cough	1	2	15.4
History of fever (>38 °C)	1	3	31.6
		4	55.7

Note:

Table modified from Aalbers et al. [7]*.*

Scarlet fever is mainly a clinical diagnosis made through the history and examination. However, in cases where there is diagnostic doubt, a tonsillar swab can be taken [7]. This is best used with the Centor Score as it would not be feasible nor cost-effective to swab every patient who presents with these symptoms.

Because scarlet fever presents with such a wide variation in severity, it is difficult to diagnose in its early stages. A prolonged duration of pyrexia (>38.5 °C) [8] and the degree of tachycardia coupled with the spreading bilateral trunk rash, results in a confident Scarlatina diagnosis [9].

## Action to take

### Physical examination

During the physical examination, it is important to be vigilant for spreading rashes on the trunk and the medial surfaces of elbows, in addition to the desquamation of the palms. Anterior cervical node enlargement is also seen, coupled with a high temperature. When a child presents with pyrexia and tachycardia, it is important to examine the skin just below the underwear line. Other signs include enlarged papillae on the tongue (strawberry tongue) and petechiae on the soft palate.

### Treatment

The risks from these complications provide strong support for an early diagnosis and immediate treatment. Beta lactam antibiotics are the preferred treatment for GAS infection due to their clinical efficacy, safety record in children, and low cost [1]. Penicillin seems to consistently clinically outperform cephalosporins and macrolides in treating Group-A Beta Haemolytic Streptococcus. Its low cost supports its selection as a first choice treatment. Macrolides such as azithromycin produce greater adverse effects than penicillin [10].

Reports of macrolide resistance are increasing due to trends in overprescribing, which leads to an increased selection pressure for pathogenic bacteria as the drug has a long half-life and a broad spectral antibacterial action. Thus, it is important to correctly diagnose to avoid the incorrect prescription of macrolides for suspected bacterial infections when the causative agent is of viral origin [11]. It is important for patients to be informed to complete the prescribed course and contact their clinician if they have any concerns to minimise likelihood of antibiotic resistance.

Phenoxymethylpenicillin Penicillin (V) should be prescribed four times a day for ten days. Symptoms can be treated with over-the-counter drugs such as paracetamol or ibuprofen and fluid replacement [12]. Scarlet fever is a notifiable disease and centres of education such as schools and nurseries should be encouraged to report cases to Public Health England.[12,13]

## Complications

The early treatment of scarlet fever is important, both to rectify symptoms and to prevent further spread of infection [6]. The complications mostly present in the mediastinal area or arise from lymphatic or haematogenous spread. Local complications include peritonsillar and retropharyngeal abscesses, and usually present with prolonged symptoms or localised pain; affected patients generally have a toxic appearance [1,14]. Other complications include acute rheumatic fever, glomerulonephritis, bacteraemia, pneumonia, endocarditis, and meningitis [1]. Rarely, complications from scarlet fever may include hepatitis, gallbladder hydrops or splenomegaly [15]. In the case of hepatitis, jaundice may or may not be present, making diagnosis challenging. However, treatment with antibiotics yields good recovery [15]. Most of the evidence about sequelae comes from case reports from the 1950s due to a high frequency of complications in that era [13].

## Disclosure statement

No potential conflict of interest was reported by the authors.
